# Automatic breast ultrasound: state of the art and future perspectives

**DOI:** 10.3332/ecancer.2020.1062

**Published:** 2020-06-23

**Authors:** Luca Nicosia, Federica Ferrari, Anna Carla Bozzini, Antuono Latronico, Chiara Trentin, Lorenza Meneghetti, Filippo Pesapane, Maria Pizzamiglio, Nicola Balesetreri, Enrico Cassano

**Affiliations:** 1 Department of Breast Radiology, European Institute of Oncology, 20141 Milan, Italy; 2 Postgraduation School in Radiodiagnostics, Università degli Studi di Milano, 20122 Milan, Italy; 3 Department of Radiology, European Institute of Oncology, 20141 Milan, Italy

**Keywords:** automatic breast ultrasound, retraction phenomenon, clinical practice, screening

## Abstract

The three-dimensional automated breast ultrasound system (3D ABUS) is a new device which represents a huge innovation in the breast ultrasound field, with several application scenarios of great interest.

ABUS's aim is to solve some of the main defects of traditional ultrasound, such as lack of standardization, high level of skill non-reproducibility, small field of view and high commitment of physician time. ABUS has proven to be an excellent non-ionising alternative to other supplemental screening options for women with dense breast tissue; also, it has appeared to be very promising in daily clinical practice.

The purpose of this paper is to present a summary of current applications of ABUS, focusing on clinical applications and future perspectives as ABUS is particularly promising for studies involving artificial intelligence, radiomics and evaluation of breast molecular subtypes.

## Background

Nowadays, breast cancer is still characterised by high mortality: the early identification of breast pathology has always been a challenge for research studies and new devices for increasing the identifications of early-stage tumours are needed. It is well known that, in women with dense breasts, mammography is less accurate in the detection of cancer [1] and screening risks to fail the identification of potentially deadly neoplasia. Over the years, the complementary use of the hand-held ultrasound (HHUS) in daily clinical practice, and in screening for some study protocols, has led to excellent results and to better detection of breast tumours [2–4]. In spite of these achievements, HHUS presents some defects that are often difficult to solve as a lack of standardisation, high level of skill non-reproducibility, small field of view (FOV) and high commitment of physician time [3]. In this scenario, the invention of a new device, such as the automated breast ultrasound (ABUS), has tried to overcome this kind of problems [5, 6] offering a minor time of acquisition, larger FOV, high-resolution imaging, multiplanar reformations and probe designed to fit the normal curvature of the breast minimising the induced artefacts of the periphery [7]. Furthermore, ABUS allows the radiologist to interpret the images in a separate time after the acquisition, optimizing the physician time.

In many studies, ABUS has proven to be an excellent, non-ionising alternative to other supplemental screening options for women with dense breast tissue [8–10]. More controversial is the possibility of using ABUS in the daily clinical practice: in this field, there are several studies that demonstrate excellent results with ABUS, especially compared with conventional HHUS, but they have been performed on a much smaller number of patients compared to those performed to evaluate the effectiveness of ABUS in screening.

The aim of this work is to revise the reliability of the ABUS in screening and daily clinical practice. Our hope is to give a new perspective in the use of the ABUS in a broader context [11, 12]. This new scenario would potentially offer excellent results, especially for the management of patients.

## Technique

The ABUS is a computer-based system for evaluating the whole breast. Each breast is imaged in three views: anteroposterior, medial, and lateral, with an automated 6–14 MHz linear array transducer attached to a rigid compression plate. This system received FDA clearance as an adjunct to screening mammography in 2008 [13].

The transducer moves automatically over the breast, in a way similar to that of HHUS, acquiring transverse images in craniocaudal linear overlapping rows.

Each of the three views is acquired up to about 300 2D images and, from all of them, it is possible to get multiplanar reconstructions of the entire breast, from the skin to the chest wall: in particular, the coronal plane, also known as the ‘surgical view’, is essential for the review phase. In fact, the standardised review process for quick navigation involves a patented thick-slice coronal plane.

Nowadays, several types of ABUS systems are available, including two main categories: prone- and supine-scanners [14].

Prone-type scanners are still under development; while supine scanners are regularly used in clinical practice.

When the examination is performed in the supine position, a towel of sponge is placed under the shoulder: this helps to spread out the breast tissue evenly, with the nipple pointing to the ceiling. A hypoallergenic lotion is placed evenly on the breast with an additional amount on the area of the nipple to allow adequate contact between the probe and the skin.

The ABUS scan is continuous and automated. During the acquisition, women are asked not to move and to breathe smoothly. Volume acquisitions are obtained in the axial plane starting from the inferior part of the breast with coronal and sagittal reconstruction. Image data automatically acquire a 15.4 cm × 17.0 cm volume from the skin to the chest wall up to 5 cm deep with a 0.2-mm thickness of each slice.

For each breast, three volumes are obtained: the central (anteroposterior) volume with the nipple in the centre of the footprint (shape of a donut), the lateral volume that included the upper outer part of the breast tissue with the nipple located in the inferior-medial corner and the medial volume that included the inner and inferior part of the breast tissue. A nipple marker is placed in every examination for accurate localisation of different quadrant of the breast.

The depth of the scan should be evaluated to ensure that the deep and the peripheral breast tissues are included in the image fields depending on the breast size: from 3.5 to 5 cm, respectively, for small, medium and large breasts. For optimal image quality, in women with larger breasts, additional views (superior and inferior) are taken to avoid the upper and deep central tissue exclusion.

Conventionally, three 1 minute scans are enough for scanning the entire breast, excluding the axilla. However, in the case of larger breasts, more than one scan may be required to cover the entire field of interest. The average total time to complete the examination is approximately 15 minutes.

Afterimage acquisition by the technicians, the data are saved and transferred to dedicated workstations where radiologists, in a separate time, can review them using both native and reconstructed scans.

Hence, ABUS increases reproducibility, reduces operator-dependence and physicians time due to the possibility to review images retrospectively, and adds new diagnostic information with multiplanar reconstructions. On the other hand, there are also some main limitations, such as the exclusion of axillary regions from the field of view and the absence of tools to assess vascularity and tissue elasticity [14].

**Figure 1.**
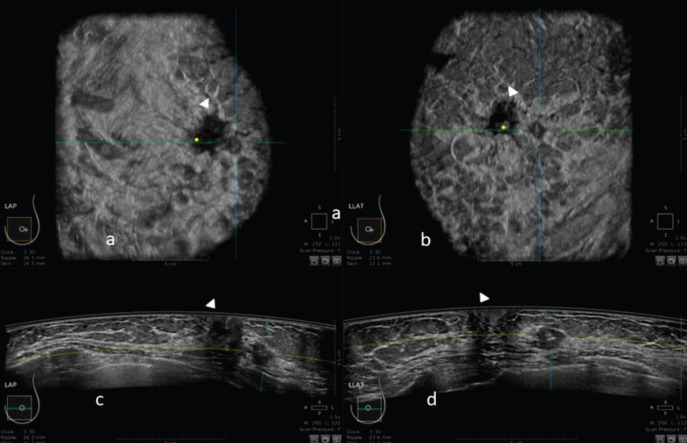
3D ultrasound image at the dedicated workstation of a 82-year-old patient with biopsy proven ductal infiltrating carcinoma. a and b: reconstructed coronal plane; the lesion is marked as a reference point. Arrowhead shows the nipple. c and d: axial plane: the lesion is marked as a reference point. Arrowhead shows the nipple.

An example of the ABUS image is shown in Figure 1.

## Applications

### Screening

The use of an ABUS designed for breast cancer screening was proposed by some authors [5, 6] already since 1980, in order to overcome the limitations of mammography. Interest in ABUS’s application for screening has further grown, especially in recent years, due to increased awareness among the problem of breast [15].

The application of ABUS as a complementary screening technique has been widely consolidated in the literature for women with dense breast indeed. There are some studies in the literature, concerning a large number of patients, with very numerous populations, some mono and others multicentric, which all agreed that the combined use of mammography and ABUS in screening women with dense breasts lead to an augmented sensitivity in breast cancer detection with a not significant reduction in specificity, as reported in Table 1.

Kelly *et al* [8] published a multicentre study based on 4,419 women with dense breasts and/or at elevated risk of breast cancer, compared the diagnostic performance of mammography alone versus that of ABUS plus mammography. The results shown that the mean sensitivity increased from 50% to 81%, an improvement of 63% in the number of cancer cases identified: all the readers involved in the study found more cancers individually, and all found 16%–29% more cancers than the best mammography reader did with mammography alone. Specificity was 89.9% for ABUS, 95.15% for mammography and 98.7% for the combined modalities [8]. Kelly *et al* [16] have also conducted a study on radiologists’ performance in detecting lesions in dense breasts using mammography alone versus automated whole breast ultrasound plus mammography: also in this case the sensitivity increased by adding ABUS, from 50% to 81%. Noteworthy, the interpretation time per ABUS was 7 minutes 58 seconds, shorter than the time reported in the ACRIN 6666 trial regarding HHUS screening (19 minutes).

Giuliano [17] in a study performed in 3,418 asymptomatic women with mammographically dense breasts shows a detection of mammography plus ABUS of 12.3 per 1,000 breast cancers, compared to 4.6 per 1,000 by mammography alone.

The Somoinsight study [7] in 2015, a multicentre prospective trial, including 15,318 asymptomatic women with dense breast, compared mammography versus mammography plus ABUS. As results, combined imaging approach led to an increase in cancer detection rate of 1.9 per 1,000 women with a rise in sensitivity of 26.7% and specificity of 85.4% for mammography alone versus 72% for mammography plus ABUS.

Even Wilczek *et al* [9] in 2016 , in a single-centre study, evaluated 1,668 asymptomatic women, with heterogeneously dense (50%–74% dense tissue) / extremely dense (³75% dense tissue) breast parenchyma. The combination of digital mammography plus ABUS determined an increase in cancer detection of 2.4 per 1,000 women screened. The increase in sensitivity was 36.4% for combined modalities versus mammography alone at study entry, while, including interval cancers, sensitivity increased by 25%. Specificity decreased by 0.7% when ABUS was added to mammography.

Giger *et al* [10], in a multi-reader study on asymptomatic women with BIRADS C or D breast density, shown an improvement in detection of both mammography-negative and mammography-positive breast cancers with the use of ABUS. The improvement in sensitivity was 23.9%, for mammography-negative breast cancers (*p* = 0.004) and 5.9% for mammography-positive breast cancers (*p* = 0.234); specificity decreased from 78.1% for mammography alone to 76.2% for the combined modalities. Combined ABUS-mammography compared to mammography alone, significantly improved reader’s detection of breast cancers in women with dense breast tissue without substantially affecting its specificity.

Those results can be further improved with more radiologists’ experience and confidence in the new method especially by reducing unnecessary recall [18].

Indeed, the adjunct of ABUS to screening mammography increased the recall rates and doubled the recalls leading to biopsy, with a decrease in positive predictive value.

The results of screening applications of ABUS are summarised in Table 1.

## Clinical practice

ABUS, compared to HHUS, is still under examinations in different clinical aspects: detection rate and characterisation of breast lesions, diagnostic performance, sensibility and specificity, inter-observer agreement and use in the pre-operative setting or as a second look procedure.

There are several studies [19–22] although performed in relatively small numbers of patients, that demonstrate similar results in terms of sensitivity and specificity of ABUS compared to HHUS, as it is shown in Table 2.

Considering the studies with the largest number of patients: Wang *et al* [19], in a study of 213 patients reported a sensitivity of ABUS versus HHUS of 95.6% versus 90.3% and a specificity of 80.5% versus 82.5%. Jeh *et al* [23], in a study of 173 patients reported a sensitivity of 88% versus 95.7% and a specificity of 76.2% versus 49.4%.

More recently, Lin Niu *et al* [24] in 2019 studied 599 breast lesions in 398 women comparing ABUS and HHUS with pathologic results or 1-year follow-up as a reference. The results shown that there was a significant difference between ABUS and HHUS in terms of sensitivity (92.23% versus 82.52%; *p* < 0.01) but not in terms of diagnostic accuracy, specificity, positive and negative predictive value.

Also, as expected, the detection rate increases as the lesion size increases [25]. Anyway, only few studies have obtained a statistically significant increase in the detection rate of ABUS compared to HHUS: those of Zhang *et al* [26], Xiao *et al* [27] (Table 3).

Even in terms of characterisation capacity of a breast lesion according to the BIRADS classification, ABUS and HHUS are similar. Examples are the studies of Golatta *et al* [28], which found good agreement between ABUS and HHUS (*k* = 0.34) in assigning a BIRADS value: in particular, this agreement increases (*k* = 0,68) if you characterise a lesion by assigning it to two categories (non-suspect = BIRADS 1–2 or suspect = BIRADS 4–5). Similar results in terms of concordance of assignment of a BIRADS value were obtained in the studies of Shin *et al* [25] (*k* = 0,64) and Kim *et al* [29] (*k* = 0.773 ± 0.104). In Kim’s study, the BIRADS descriptor that presented the best agreement was the ‘orientation’ (*k* = 0.608 ± 0.210) and the worst was the ‘posterior echo feature’ (*k* = 0.371 ± 0.225); Kotsianos-Hermle *et al* [30] reported a good correlation for the descriptor ‘margin’.

Consistent with the good characterisation of breast lesions, ABUS has good diagnostic performances in differentiate benign and malignant lesions without significant superiority or inferiority over HHUS, in the majority of studies [19, 20, 22, 30–33]. However Choi *et al* [34], in a study conducted in 5,566 asymptomatic women, 1,866 evaluated with ABUS and 3,700 with HHUS, demonstrated a significant difference between ABUS and HHUS, in terms of diagnostic accuracy (97.70% of ABUS versus 96.54% of HHUS; *p* = 0.022) and specificity (86.2% versus. 87.5%; *p* = 0.018); no difference in terms of sensitivity was observed (77.78% versus 62.50%; *p* > .05). Similar data of diagnostic accuracy are observed in the study of Kim *et al* [29].

More recently, Zhang *et al* [35] demonstrated a better diagnostic performance of ABUS versus HHUS, in particular in the detection of precancerous lesions or cancers (BIRADS 4-5). In this hospital-based multicentre diagnostic study, Zhang *et al* [35] have evaluated the clinical performance of the ABUS for breast cancer detection by comparing it to handheld ultrasound and mammography (MG) in 1973 women, from 30 to 69 years. The results not only shown a good diagnostic performance of ABUS but also that the ABUS results, compared to HHUS, were more consistent with the pathology results in the BIRADS 4–5 groups: 78.6% of women classified as BI-RADS 4–5 based on the ABUS were diagnosed with precancerous lesions or cancer, which was 7.2% higher than that of women based on HHUS. For BI-RADS 1–2, the false-negative rates of the ABUS and HHUS were almost identical and were much lower than those of MG.

Furthermore, it appears that ABUS has shown a better ability to identify calcifications and the ‘retraction phenomenon’, a new diagnostic information peculiar of coronal plane reconstruction, with high accuracy for malignant breast lesions: Lin *et al* [21] have achieved an accuracy of 91.4%.

Vourtsis *et al* [36] shown that ABUS outperform HHUS in the detection of architectural distortion as ‘a retraction phenomenon sign’ due to the coronal plane and can support mammography in the detection of non-calcified carcinomas in women with dense breasts.

The retraction phenomenon is defined as the convergence tendency of the tissue surrounding a lesion with or without cord-like hyperechogenicity intervals on the coronal plane. Its most important clinical application is the high specificity for breast malignancies: in malignant masses, retraction phenomenon is more commonly seen in tumours of small size, short distance to the skin and low histological grade [36].

To get a significant impact of ABUS on clinical practice, a good inter-observer reliability is mandatory. The studies in the literature have tested the agreement between different readers in terms of allocation of a BIRADS category and description of the characteristics of breast lesions (such as location, size, shape, etc..): the results are heterogeneous but the agreement between readers may increase with a dichotomization of BIRADS score in two categories (non-suspect = BIRADS 1–2 or suspect = BIRADS 4–5) [37–39].

**Figure 2.**
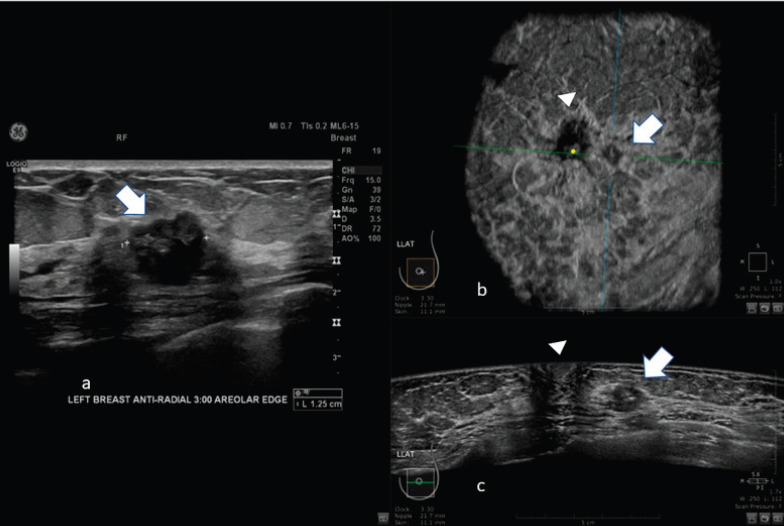
62-year-old patient with biopsy proven left breast carcinoma. a: Hand-held ultrasound showing the lesion (arrow). b: 3D ultrasound (ABUS) image at the dedicated workstation. Reconstructed coronal plane: the lesion is marked with the arrow; the nipple is marked with the arrowhead. c: reconstructed axial plane of Automatic Breast Ultrasound (ABUS): the lesion is marked with with the arrow; the nipple is marked with arrowhead.

**Figure 3.**
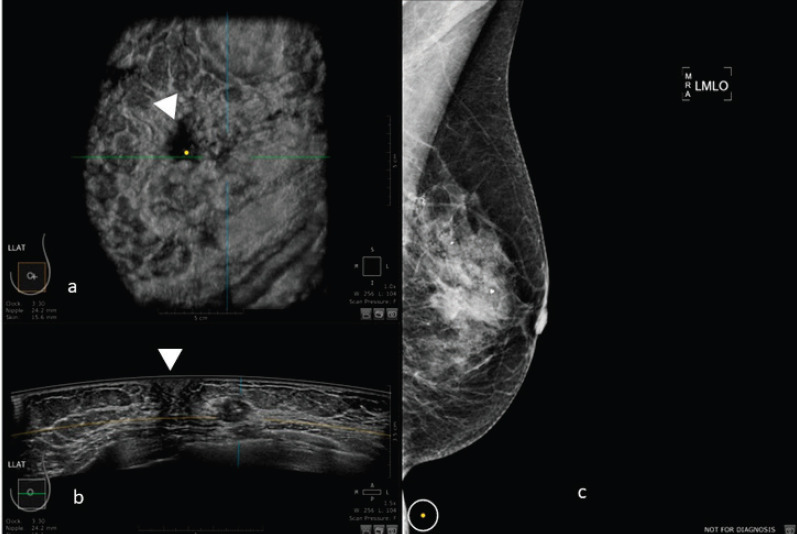
50-year-old patient with biopsy proven left breast carcinoma. a: 3D ultrasound (ABUS) image at the dedicated workstation. Reconstructed coronal plane: the lesion is marked as a reference point. The nipple is marked with the arrowhead. b: 3D ultrasound (ABUS) image at the dedicated workstation. Reconstructed axial plane: the lesion is marked as a reference point. The nipple is marked with the arrowhead. c: Full field digital mammography of the left breast in which the lesion is fairly visible.

Images of comparison between ABUS, HHUS and mammography are shown in Figures 2 and 3.

In conclusion, the great majority of the studies using the new-generation ABUS scanners reported high sensitivity and specificity, comparable or sometimes better than HHUS and full field digital mammography (FFDM).

ABUS avoids the dependence on the sonographer and allows to standardize the examination procedure.

Surely, we have to consider some weak points: the importance of the learning curve, as limited experience with ABUS, can affect sensitivity and specificity, the inability of ABUS to evaluate the axillary region, to investigate lesion vascularization of tissue elasticity.

## Artifacts

The effectiveness of ABUS has been demonstrated in many studies [19, 20, 22, 30–33], however many artifacts can reduce diagnostic appropriateness.

Corrugation is the most common artifacts: it is due to respiratory motion.

It is essential that during the examination the patient avoid to speak or to cough [40].

Similarly, it is important to ensure an uniform compression and proper position of the breast and to avoid insufficient lotion application: indeed one important artifact it’s the posterior shadowing that develops at the interface of fat lobules, due to the lack of previous precautions [41].

## Other applications

Some studies have also evaluated the effectiveness of ABUS compared to HHUS in pre-operative cancer assessment. ABUS was more accurate than HHUS in assessing the extent of the disease and the mean lesion size [12], the larger diameter [42] and the total volume [43] being essential in the preoperative field, to assess the real extension of the lesion.

Furthermore, ABUS seems to be excellent as a ‘second look’ tool after breast MRI (reference needed).

This can be very useful for the low specificity of MRI.

**Figure 4.**
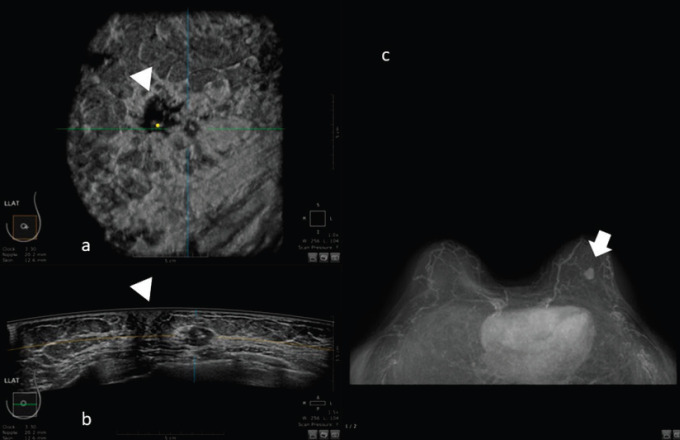
50-year-old patient with biopsy proven left breast carcinoma. a: 3D ultrasound (ABUS) image at the dedicated workstation. Reconstructed coronal plane: the lesion is marked as a reference point. The nipple is marked with the arrowhead. b: 3D ultrasound (ABUS) image at the dedicated workstation. Reconstructed axial plane: the lesion is marked as a reference point. c: Breast MRI: the lesion is marked with the arrow.

For example, in the study of Schmachtenberg [44], 3D ABUS correlates well with MRI and histopathological measurements. An example of this correlation is shown in Figure 4.

Chae *et al* [45] shown that on 729 MRI, 80 additional suspicious lesions in 58 patients (age range: 27–63 years, mean: 45 years) were identified by pre-operative MRI. Of the 80 suspicious lesions detected at MRI in 58 women, ABUS detected 70/80 lesions, 10% more than HHUS. Automated breast ultrasound can reliably detect additional suspicious lesions that have been identified on breast MRI being more rapid and less costly than HHUS and may help in the decision on the biopsy guidance method (HHUS versus MRI).

These data were also confirmed by Kim *et al* [46] through a prospective study in which they found 76 suspicious lesions in 40 women who underwent pre-operative MRI. ABUS had significantly higher values of detection rates than HHUS (94.7% versus 86.8%; *p* < 0.05).

Another interesting application is the possibility to correlate the imaging features obtained by an ABUS and molecular subtypes of breast cancer:

Zheng *et al* [47] in a study on 303 malignant breast tumours shows a strong correlation between the “retraction phenomenon” and the molecular subtypes.

Similar results were obtained by Wang *et al* [48] in a study performed on 118 lesions and by Jiang *et al* [49] in a study performed on 85 patients.

**Figure 5.**
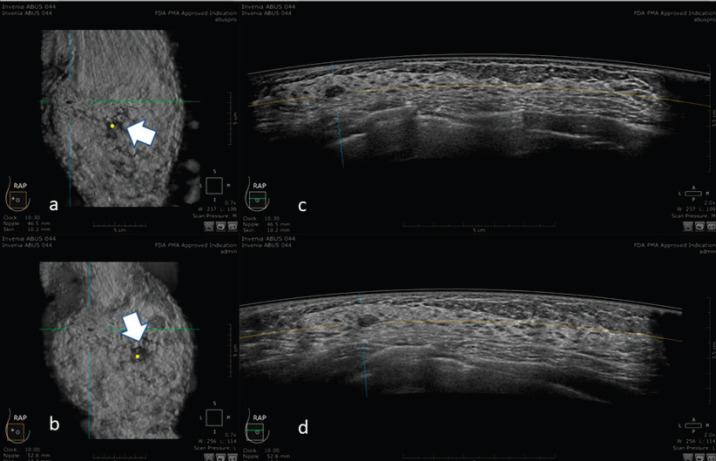
30-year-old patient with right breast carcinoma: a and b: 3D ultrasound (ABUS) image at the dedicated workstation. Reconstructed sagittal plane: the lesion is marked as a reference point. The nipple is marked with the arrow. c and d: 3D ultrasound (ABUS) image at the dedicated workstation. Reconstructed axial plane: the lesion is marked as a reference point.

Compared to HHUS, ABUS is therefore a valuable additional tool not only in screening but also in the clinical field, in the characterisation and diagnostic accuracy of breast lesions, as reported in Figure 5.

### Artificial intelligence

For its reproducibility and way of preserving images, the ABUS lets the use of computer-aided design CAD [50] and techniques of artificial intelligence aimed at increasing diagnostic performance with deep machine learning: for example Block-Based Region Segmentation, can be done relatively easily with ABUS compared to conventional HHUS allowing the use of traditional machine learning models. ABUS opens promising scenarios in the field of artificial intelligence to be confirmed with other studies. Future perspectives include the integration of radiomics and deep learning in the further development of 3D ABUS [51].

Indeed, Radiomic 3D ABUS signature, combinations of imaging features, could accurately differentiate between malignant and benign breast lesions [52].

## Conclusions

The ABUS offers valuable impact in the detectability of breast lesions and the differentiation of malignant from benign lesions, with a higher inter-observer agreement. Its use, both in screening, in addition to mammography, and in clinical practice, seems to offer excellent results, not inferior to those of traditional ultrasound, with the advantage of great saving in medical time and in reducing some of the most common problems of the HHUS.

Furthermore, due to its characteristics, ABUS is particularly promising for studies involving artificial intelligence and radiomics, opening new extremely interesting study scenarios in the diagnostic field.

## Conflicts of interest statement

All authors have no conflicts of interest.

## Funding statement

This research did not receive any specific grant from funding agencies in the public, commercial, or not-for-profit sectors.

## Figures and Tables

**Table 1.** Value of sensibility, specificity and detection rate of Full Field digital Mammography and Full field digital Mammography plus Automatic Breast Ultrasound in screening programme.

Author	Year	Nb of pt	Sensibility %		Specificity %		Detection rate per 1,000	
			FFDM	FFDM plus ABUS	FFDM	FFDM plus ABUS	FFDM	FFDM plus ABUS
Kelly *et al* [16]	2010	4419	40	81	95.15	98.7	2,6	6,5
Giuliano *et al* [17]	2012	3418	76,00	96.7	99.70	98.2	4,6	12,3
Brem (The Somoinsight Study) [7]	2014	15318	73.2	100	85.4	72	5,4	7,3
Wilczek *et al* [9]	2016	1668	63.6	100	99	98.4	4,2	6,6
Giger *et al* [10]	2016	185	57.5	74.1	78.1	76.2	//	//

FFDM: Full field digital mammography.

ABUS: Automatic breast ultrasound.

Nb of pt: number of patients.

**Table 2.** Comparison of sensibility and specificity of ABUS (Automatic Breast Ultrasound) and US (Hand Held Ultrasound) in daily clinical practice.

Author	Year	Number of patients	Sensibility %		Specificity %	
			ABUS	US	ABUS	US
Cho *et al* [33]	2006	141	98.3	96.7	96.7	64.4
Kotsianos-Hermle *et al* [30]	2009	97	96,5	97.5	923	88.5
Shin *et al* [25]	2011	55	96	100	91,8	93
Chang *et al* [32]	2011	67	92	//	63	//
Wang *et al* ^19^	2012	213	95.3	90.6	80.5	82.5
Lin *et al* [21]	2012	81	100	95	100	85
Wang *et al* [20]	2012	155	96.1	93.2	91.9	88,7
Chen *et al* [31]	2013	175	92.5	88.1	86.2	87.5
Kim *et al* [22]	2013	38	92	98	87.5	62.5
Kim *et al* [29]	2014	87	89.2	98.7	79	80.1
Jeh *et al* [23]	2016	173	88	95.7	76.2	49.4
Schmachtenberg *et al* [44]	2017	28	93.3	100	83.3	83.3
Niu *et al* [24]	2019	398	92.23	82.52	77.62	80.24

**Table 3.** Studies in which the detection rate of ABUS (Automatic Breast Ultrasound) is significantly better of US (hand held ultrasound) in clinical practice.

Author	Year	Number of patients	Detection %	
			ABUS	US
Zhang *et al* [26]	2012	82	89.9	60.6
Xiao *et al* [27]	2015	300	100	78.2

## References

[1] Kolb TM, Lichy J, Newhouse JH (2002). Comparison of the performance of screening mammography, physical examination, and breast US and evaluation of factors that influence them: an analysis of 27,825 patient evaluations. Radiology.

[2] Hooley RJ, Greenberg KL, Stackhouse RM (2012). Screening US in patients with mammographically dense breasts: initial experience with Connecticut public act 09-41. Radiology.

[3] Scheel JR, Lee JM, Sprague BL (2015). Screening ultrasound as an adjunct to mammography in women with mammographically dense breasts. Am J Obstet Gynecol.

[4] Berg WA, Blume JD, Cormack JB (2008). Combined screening with ultrasound and mammography vs mammography alone in women at elevated risk of breast cancer. JAMA.

[5] Maturo VG, Zusmer NR, Gilson AJ (1980). Ultrasound of the whole breast utilizing a dedicated automated breast scanner. Radiology.

[6] Bassett LW, Kimme-Smith C, Sutherland LK (1987). Automated and hand-held breast US: Effect on patient management. Radiology.

[7] Brem RF, Tabár L, Duffy SW (2015). Assessing improvement in detection of breast cancer with three-dimensional automated breast US in women with dense breast tissue: the somoinsight study. Radiology.

[8] Kelly KM, Dean J, Comulada WS (2010). Breast cancer detection using automated whole breast ultrasound and mammography in radiographically dense breasts. Eur Radiol.

[9] Wilczek B, Wilczek HE, Rasouliyan L (2016). Adding 3D automated breast ultrasound to mammography screening in women with heterogeneously and extremely dense breasts: Report from a hospital-based, high-volume, single-center breast cancer screening program. Eur J Radiol.

[10] Giger ML, Inciardi MF, Edwards A (2016). Automated breast ultrasound in breast cancer screening of women with dense breasts: reader study of mammography-negative and mammography-positive cancers. Am J Roentgenol.

[11] Chang JM, Cha JH, Park JS (2015). Automated breast ultrasound system (ABUS): Reproducibility of mass localization, size measurement, and characterization on serial examinations. Acta Radiol.

[12] Li N, Jiang YX, Zhu QL (2013). Accuracy of an automated breast volume ultrasound system for assessment of the pre-operative extent of pure ductal carcinoma in situ: comparison with a conventional handheld ultrasound examination. Ultrasound Med Biol.

[13] Gazhonova V (2017). 3D automated 39breast volume sonography. 3D Automated Breast Volume Sonography.

[14] Kaplan SS (2014). Automated whole breast ultrasound. Radiol Clin North Am.

[15] Durand MA, Hooley RJ (2017). Implementation of whole-breast screening ultrasonography. Radiol Clin North America.

[16] Kelly KM, Dean J, Lee SJ (2010). Breast cancer detection: Radiologists’ performance using mammography with and without automated whole-breast ultrasound. Eur Radiol.

[17] Giuliano V, Giuliano C (2013). Improved breast cancer detection in asymptomatic women using 3D-automated breast ultrasound in mammographically dense breasts. Clin Imaging.

[18] Arleo EK, Saleh M, Ionescu D (2014). Recall rate of screening ultrasound with automated breast volumetric scanning (ABVS) in women with dense breasts: a first quarter experience. Clin Imaging.

[19] Wang HY, Jiang YX, Zhu QL (2012). Differentiation of benign and malignant breast lesions: a comparison between automatically generated breast volume scans and handheld ultrasound examinations. Eur J Radiol.

[20] Wang ZL, Xw JH, Li JL (2012). Comparison of automated breast volume scanning to hand-held ultrasound and mammography. Radiol Med.

[21] Lin X, Wang J, Han F (2012). Analysis of eighty-one cases with breast lesions using automated breast volume scanner and comparison with handheld ultrasound. Eur J Radiol.

[22] Kim SH, Kang BJ, Choi BG (2013). Radiologists’ performance for detecting lesions and the interobserver variability of automated whole breast ultrasound. Korean J Radiol.

[23] Jeh SK, Kim SH, Choi JJ (2016). Comparison of automated breast ultrasonography to handheld ultrasonography in detecting and diagnosing breast lesions. Acta Radiol.

[24] Niu L, Bao L, Zhu L (2019). Diagnostic performance of automated breast ultrasound in differentiating benign and malignant breast masses in asymptomatic women: a comparison study with handheld ultrasound. J Ultrasound Med.

[25] Shin HJ, Kim HH, Cha JH (2011). Automated ultrasound of the breast for diagnosis: Interobserver agreement on lesion detection and characterization. Am J Roentgenol.

[26] Zhang Q, Hu B, Hu B (2012). Detection of breast lesions using an automated breast volume scanner system. J Int Med Res.

[27] Xiao YM, Chen ZH, Zhou QC (2015). The efficacy of automated breast volume scanning over conventional ultrasonography among patients with breast lesions. Int J Gynecol Obstet.

[28] Golatta M, Franz D, Harcos A (2013). Interobserver reliability of automated breast volume scanner (ABVS) interpretation and agreement of ABVS findings with hand held breast ultrasound (HHUS), mammography and pathology results. Eur J Radiol.

[29] Kim H, Cha JH, Oh HY (2014). Comparison of conventional and automated breast volume ultrasound in the description and characterization of solid breast masses based on BI-RADS features. Breast Cancer.

[30] Kotsianos-Hermle D, Hiltawsky KM, Wirth S (2009). Analysis of 107 breast lesions with automated 3D ultrasound and comparison with mammography and manual ultrasound. Eur J Radiol.

[31] Chen L, Chen Y, Diao XH (2013). Comparative study of automated breast 3-d ultrasound and handheld b-mode ultrasound for differentiation of benign and malignant breast masses. Ultrasound Med Biol.

[32] Chang JM, Moon WK, Cho N (2011). Radiologists’ performance in the detection of benign and malignant masses with 3D automated breast ultrasound (ABUS). Eur J Radiol.

[33] Cho N, Moon WK, Cha JH (2006). Differentiating benign from malignant solid breast masses: Comparison of two-dimensional and three-dimensional US. Radiology.

[34] Choi WJ, Cha JH, Kim HH (2014). Comparison of automated breast volume scanning and hand-held ultrasound in the detection of breast cancer: an analysis of 5,566 patient evaluations. Asian Pacific J Cancer Prev.

[35] Zhang X, Lin X, Tan Y (2018). A multicenter hospital-based diagnosis study of automated breast ultrasound system in detecting breast cancer among Chinese women. Chinese J Cancer Res.

[36] Vourtsis A, Kachulis A (2018). The performance of 3D ABUS versus HHUS in the visualisation and BI-RADS characterisation of breast lesions in a large cohort of 1,886 women. Eur Radiol.

[37] Meng Z, Chen C, Zhu Y (2015). Diagnostic performance of the automated breast volume scanner: a systematic review of interrater reliability/agreement and meta-analysis of diagnostic accuracy for differentiating benign and malignant breast lesions. Eur Radiol.

[38] Wojcinski S, Farrokh A, Hille U (2011). The automated breast volume scanner (ABVS): Initial experiences in lesion detection compared with conventional handheld b-mode ultrasound: a pilot study of 50 cases. Int J Womens Health.

[39] Wenkel E, Heckmann M, Heinrich M (2008). Automated breast ultrasound: lesion detection and BI-RADS^TM^ classification—a pilot study. RoFo Fortschritte auf dem Gebiet der Rontgenstrahlen und der Bildgeb Verfahren.

[40] Xiao Y, Zhou Q, Chen Z (2015). Automated breast volume scanning versus conventional ultrasound in breast cancer screening. Acad Radiol.

[41] Karst I, Henley C, Gottschalk N (2019). Three-dimensional automated breast us: facts and artifacts. Radiographics.

[42] Huang A, Zhu L, Tan Y (2016). Evaluation of automated breast volume scanner for breast conservation surgery in ductal carcinoma in situ. Oncol Lett.

[43] Xu C, Wei S, Xie Y (2016). Three-dimensional assessment of automated breast volume scanner compared with handheld ultrasound in pre-operative breast invasive ductal carcinomas: a pilot study of 51 cases. Ultrasound Med Biol.

[44] Schmachtenberg C, Fischer T, Hamm B (2017). Diagnostic performance of automated breast volume scanning (abvs) compared to handheld ultrasonography with breast MRI as the gold standard. Acad Radiol.

[45] Chae EY, Shin HJ, Kim HJ Diagnostic performance of automated breast ultrasound as a replacement for a hand-held second-look ultrasound for breast lesions detected initially on magnetic resonance imaging. Ultrasound Med Biol.

[46] Kim Y, Kang BJ, Kim SH (2016). Prospective study comparing two second-look ultrasound techniques. J Ultrasound Med.

[47] Zheng FY, Lu Q, Huang BJ (2017). Imaging features of automated breast volume scanner: Correlation with molecular subtypes of breast cancer. Eur J Radiol.

[48] Wang XL, Tao L, Zhou XL (2016). Initial experience of automated breast volume scanning (ABVS) and ultrasound elastography in predicting breast cancer subtypes and staging. Breast.

[49] Jiang J, Chen YQ, Xu YZ (2014). Correlation between three-dimensional ultrasound features and pathological prognostic factors in breast cancer. Eur Radiol.

[50] Lo C, Shen YW, Huang CS (2014). Computer-aided multiview tumor detection for automated whole breast ultrasound. Ultrason Imaging.

[51] Liao W-X, He P, Hao J (2019). Automatic identification of breast ultrasound image based on supervised block-based region segmentation algorithm and features combination migration deep learning model. IEEE J Biomed Heal Informatics.

[52] Papanikolaou N, Vourtsis A (2018). The performance of Radiomic ABUS signature in the differentiation of benign from malignant breast lesions (Accepted Abstract).

